# De novo assembled nuclear, chloroplast, and mitochondrial genomes show high intraspecific variation in the tropical rainforest species *Symphonia globulifera*

**DOI:** 10.1093/g3journal/jkaf208

**Published:** 2025-09-10

**Authors:** Sanna Olsson, Rocío Bautista, M Gonzalo Claros, Myriam Heuertz, Ivan Scotti

**Affiliations:** Instituto de Ciencias Forestales (ICIFOR-INIA), Consejo Superior de Investigaciones Científicas (CSIC), Madrid E-28040, Spain; Plataforma Andaluza de Bioinformática, Universidad de Málaga, Málaga E-29590, Spain; Instituto de Hortofruticultura Subtropical y Mediterránea (IHSM-UMA-CSIC), Málaga E-29010, Spain; Department of Molecular Biology and Biochemistry, Universidad de Málaga, Málaga E-29071, Spain; University. Bordeaux, INRAE, BIOGECO, Cestas F-33610, France; INRAE, UR629 URFM, Ecologie des Forêts Méditerranéennes, Avignon Cedex 9 F-84914, France

**Keywords:** tropical trees, genome assembly, genomic diversity, organelles, markers, phylogeny

## Abstract

*Symphonia globulifera* (Clusiaceae) has emerged as a model organism in tropical forest ecology and evolution due to its significant ecological role and complex biogeographical history. Originating from Africa, this species has independently colonized Caribbean, Central, and South America three times, becoming a key component of tropical ecosystems across these regions. Despite the ecological importance of *S. globulifera* and other tropical tree species, our understanding of their genomic architecture remains limited compared to temperate species. To bridge this gap, we present a comparative analysis of two de novo assembled nuclear genomes of *S. globulifera*—one from a South American individual and one from an African individual—and report newly assembled chloroplast and mitochondrial genomes. Initial assembly of the organelles was performed using GetOrganelle, and the results were compared with corresponding publicly available sequences from closely related *Garcinia* species. Our study introduces novel genomic resources, including an annotated nuclear draft genome based on Illumina short reads, the first chloroplast genome assembly for the genus, and a set of assembled mitochondrial gene sequences. Additionally, we provide a set of single-copy nuclear gene alignments identified by BUSCO as well as manually curated coding plastid genes, which will serve as valuable tools for future comparative analyses and phylogenetic studies. Our preliminary results based on chloroplast genes and limited sampling suggest that *Garcinia* might be nonmonophyletic. The detected differences in nuclear and organellar genomes reveal high intraspecific variation, emphasizing the importance of genome-wide sampling for understanding tropical tree evolution.

## Introduction

With hyperdiverse tropical ecosystems facing important threats to their conservation (Barlow et al. 2018; Ferrer-Paris et al. 2019) and eco-evolutionary processes, e.g., speciation and evolutionary change, being highly variable in tropical trees depending on population sizes and spatiotemporal environmental turnover (Allié et al. 2015; Pennington and Lavin 2016), it is important to study them in more detail. However, our understanding of eco-evolutionary processes in tropical tree species lags far behind that of temperate species (Holliday et al. 2017; De Kort et al. 2021). This is particularly true for the availability of genomic resources, essential for conservation genetics studies (Formenti et al. 2022; Heuertz et al. 2023; Theissinger et al. 2023).

Plant genome assemblies typically focus on the nuclear genome, which encodes the vast majority of functional genes (Heslop-Harrison and Schwarzacher 2011). However, chloroplast and mitochondrial genomes also play essential roles in plant physiology and evolution. Chloroplasts are central to photosynthesis and are widely used in plant phylogenetics due to their relatively conserved structure and uniparental inheritance (Daniell et al. 2016). Mitochondria are involved in respiration and cellular energy production, and mitochondrial genes can provide complementary phylogenetic signals and insights into cytoplasmic inheritance (O’Conner and Li 2020). Compared to nuclear genomes, organellar genomes differ markedly in size, structure, and copy number: nuclear genomes are large and complex (typically hundreds of Mb to several Gb); chloroplast genomes are small (∼120 to 160 kb), highly conserved, and present in hundreds to thousands of copies per cell; and mitochondrial genomes are highly variable in size and structure (∼200 to 2,400 kb in plants) and often fragmented or rearranged (Sloan et al. 2012; Daniell 2016). Mitochondrial genomes are more difficult to assemble than chloroplasts due to their bigger size and high structural variability (Sloan 2013). These differences present specific challenges and opportunities for genome assembly and comparative genomics.

*Symphonia globulifera* (Clusiaceae) has become a model organism in tropical forest ecology and evolution, due to its ecological importance and intriguing distribution pattern (Dick and Heuertz 2008; Torroba-Balmori et al. 2017; Schmitt et al. 2021). With an origin in Africa, the species has spread to the Caribbean, Central, and South America three independent times (Dick et al. 2003). It occurs in a variety of environments, and as a highly abundant species, it plays a pivotal role in the maintenance of forest ecosystems and serves as a resource for numerous human activities, including timber and traditional medicinal use (Oyen 2005; ter Steege et al. 2006).

Previous studies that used SNP or SSR markers in target regions have revealed high levels of intraspecific genetic diversity in *S. globulifera* (Dick and Heuertz 2008; Torroba-Balmori et al. 2017; Schmitt et al. 2021). However, genome-scale comparisons across continents are still lacking. While a draft nuclear genome was previously assembled from an African individual (Olsson et al. 2017), genomes from other parts of its range are missing. Similarly, although chloroplast or mitochondrial genomes have been assembled for several *Garcinia* species (Wee et al. 2023), no chloroplast or mitochondrial genomes are available for *Symphonia* or other Clusiaceae outside *Garcinia*. Chloroplast genomes available from the most closely related families within the same order, Malphigiales, are in Podostemaceae (Bedoya et al. 2019) and in *Hypericum perforatum* (Liu et al. 2023). The only available mitochondrial genome from Clusiaceae is that of *Garcinia mangostana* (Wee et al. 2022). The lack of these genomic resources limits our ability to understand both the evolutionary relationships within the family and the extent of genomic differentiation within the species.

The goals of our study are (i) to assemble and annotate the nuclear, chloroplast, and mitochondrial genomes of a South American individual of *S. globulifera*; (ii) to compare these assemblies with existing genomic resources from an African individual and closely related species; and (iii) to assess intraspecific genetic variation in the species. By provinding novel, much-needed genomic resources for this tropical species, including an annotated nuclear draft genome, the first chloroplast assembly for the genus, and a set of single-copy nuclear genes and manually curated coding plastid gene sets, our study delivers resources for tropical plant genomics, useful for comparative analyses and phylogenies. The observed differences in gene content and sequence diversity between African and South American individuals highlight the substantial intraspecific variation present in the species and underscore the need for multiple reference genomes per species to represent the genetic complexity in tropical trees.

## Material and methods

### Sampling and genomic DNA sequencing

Leaves from the same *S. globulifera* individual that was used for transcriptomic sequencing by Brousseau et al. (2014) were collected in 2014. Whole genomic DNA was extracted from them using a CTAB protocol as described by Le Provost et al. (2003). Preparation of Illumina TruSeq DNA libraries and Hiseq2000 sequencing of the paired-end 100-bp libraries on the Illumina 1.8 sequencing platform was carried out by IGA Technology Services (Institute of Applied Genomics, Udine, Italy) in the same year. Qubit 2.0 Fluorometer (Invitrogen, Carlsbad, CA) was used for DNA and final library quantification. Quality of DNA was tested by Agilent 2100 Bioanalyzer High Sensitivity DNA assay (Agilent Technologies, Santa Clara, CA) prior to sequencing. Four different libraries were prepared, and two of them were repeated due to low yield of reads.

### Preprocessing

Quality of the reads was checked with FastQC before and after trimming. Trimmomatic v0.36 (Bolger et al. 2014) was used to quality filter and remove adapter sequences with the following parameters: ILLUMINACLIP:2:30:10 LEADING:3 TRAILING:3 SLIDINGWINDOW:4:15 MINLEN:50. Exact duplicates were removed with the script remove_dup_PE.py (available at https://github.com/Zhong-Lab-UCSD/MARIO) before further analyses.

Removal of chloroplast and mitochondrial reads was performed by mapping with bwa-mem against Clusiaceae organelle genomes. Only the unmapped read-pairs containing both reads were kept for nuclear genomic work.

### Chloroplast genome assembly of African individual

The reads from Olsson et al. (2017) were downloaded from https://datadryad.org/stash/dataset/doi:10.5061/dryad.78ng1. The recovery of chloroplast reads, both paired-end and mate-pair, was performed by SeqTrimNext (Falgueras et al. 2010). Using the chloroplast organelle genomes database of NCBI, the assembly was carried out using the A5 pipeline (Coil et al. 2015) and the SOAPdenovo (Xie et al. 2014 ) assembly. These protocols were selected because only long mate-pair reads were available for this individual, which limited the use of organelle-specific short-read assemblers such as GetOrganelle.

In the first method, the A5 pipeline used both paired and mate-pair reads as input with default parameters. This pipeline consists of five steps: read cleaning by Trimmomatic and error correction with SGA's k-mer module; assembly of contigs and scaffolds with the IDBA-UD algorithm; misassembly correction via read mapping; and a final round of stringent scaffolding.

The second method, SOAPdenovo, assembled fragments and paired-end sequencing from the libraries with variable insert sizes. Read overlaps were represented using de Brujin graphs, with erroneous connections removed to generate the final graph. The results from the A5 pipeline were selected for downstream analyses due to longer scaffolds and higher N50 value.

The final scaffold derived from the A5 pipeline was mapped against the *Jatropha curcas* chloroplast genome (European Nucleotide Archive, ENA, accession number FJ695500.1, by Asif et al. (2010); see also Wee et al. 2023) using Bowtie2 v2.1 (Langmead et al. 2019) with default parameters. Mapped scaffolds were ordered and merged into one pseudochloroplast genome using Mauve (Darling et al. 2004). The assembly was annotated using GeSeq (Tillich et al. 2017, available at https://chlorobox.mpimp-golm.mpg.de/geseq.html; accessed on 18 August 2023). Four *Garcinia* species: *Garcinia gummi-gutta*, *G. mangostana*, *Garcinia oblongifolia*, and *Garcinia pedunculata* (see Table 1 for accession numbers) were used as references for the BLAST-like Alignment Tool (BLAT; Kent 2002) implemented in GeSeq.

**Table 1.** GenBank accession numbers of the full chloroplast genomes used in the phylogenetic study.

Species	Family	Accession	Size in bp
*Jatropha curcas*	Euphorbiaceae	NC_012224	163,856
*Erythroxylum novogranatense*	Erythroxylaceae	NC_030601	163,937
*Garcinia anomala*	Clusiaceae	MW582313	156,774
*Garcinia gummi-gutta*	Clusiaceae	NC_047250	156,202
*Garcinia mangostana* var. Mesta	Clusiaceae	MZ823408	156,580
*Garcinia mangostana* var. Thailand	Clusiaceae	NC_036341	158,179
*Garcinia oblongifolia*	Clusiaceae	NC_050384	156,577
*Garcinia pedunculata*	Clusiaceae	NC_048983	157,688
*Garcinia paucinervis*	Clusiaceae	MT501656	157,702
*Symphonia globulifera* (this study)	Clusiaceae	OZ172679	156,363

### Chloroplast genome assembly and annotation of American individual

GetOrganelle v1.7.7.0 (Jin et al. 2020) was used for chloroplast assembly, using all fastq reads as input. Parameters suggested for low-coverage assembly were used, i.e., -R 35, -k 13,21,33,45,65,85, embplant_pt, -w 60, and the chloroplast genome of *G. mangostana* (ENA accession number MZ823408, Wee et al. 2023) as seed. The assembly resulted in six scaffolds, of which five were selected as suggested by the program. The sixth scaffold was verified by BLAST search not to belong to the assembly before discarding it as contamination. The assembly was visualized with Bandage (Wick et al. 2015). RegCloser (Cao et al. 2023) was used to try to close the gaps in the assembly, unsuccessfully.

Manual alignment of the assembled scaffolds of both *S. globulifera* individuals, along with *G. mangostana* var. Mesta (ENA accession number MZ823408), and four RefSeq *Garcinia* chloroplasts (ENA accession numbers NC_036341, NC_047250, NC_048983 and NC_050384, see also Wee et al. 2023) was performed in PhyDE v1.0 (Müller et al. 2005). This facilitated scaffold orientation, detection of potential chimeras, and estimation of missing data. The manually curated assembly was annotated using GeSeq, in the same manner as for the African individual, with the same four *Garcinia* species as references in the BLAT step. The plastome map was visualized using the Organellar Genome DRAW (OGDRAW v1.3.1) program with default parameters (Greiner et al. 2019). The annotated chloroplast genome was submitted to ENA with accession number OZ172679.1 (Table 1).

Although different assembly protocols were used for the two individuals due to the distinct sequencing technologies (mate-pair long reads vs Illumina short reads), both assemblies were aligned to the same reference sequences, manually curated, and annotated using the same pipeline and references to ensure consistency. This strategy minimized potential biases introduced by methodological differences in downstream comparisons.

### Phylogenetic analysis of chloroplast genes

The gene regions (including exons and introns) of the 77 protein-coding genes were extracted from the GeSeq annotated assembly of the American *S. globulifera* chloroplast genome using the function GenBank Feature Extractor from Sequence Manipulation Suite (Stothard 2000). The same genes from the African *S. globulifera* chloroplast genome were retrieved from the manually curated alignment. The corresponding genes of all available *Garcinia* species, *Erythroxylum novogranatense*, and *J. curcas* (see Wee et al. 2023) were downloaded from GenBank (Table 1). The latter two were used as outgroups in the phylogenetic analysis. The individual gene alignments were concatenated with SequenceMatrix v1.8 (Vaidya et al 2011), defining *J. curcas* as an outgroup. Sequences were aligned with Mafft (Katoh et al. 2002) using alignment option FFT-NS-I. The alignments were edited manually, applying CDS adjustments reported by Wee et al. 2023 (*atpF*, *cemA*, *ndhA*, *ndhD*, *ndhE*, *ndhK*, *petD*, *psbM*, *rpoC1*, *rps12*, *rps16*, *rps19*). Repeats were identified in the noncoding regions of *petD* and *clpP*, and the alignments were adjusted according to the criteria laid out by Kelchner (2000). The *rpl16* intron was deleted in the two outgroup species due to very high variability and uncertain homology assessment. The data matrix was analyzed by maximum likelihood (ML) after automatic model selection using ModelFinder (Kalyaanamoorthy et al. 2017) implemented in IQTree v1.6.12 (Nguyen et al. 2015) applying 1000 ultrafast bootstrap replicates (Hoang et al. 2018). Consensus topology and support values were drawn using TreeGraph2 (Stöver and Müller 2010).

### Mitochondrial genome assembly and annotation

The mitochondrial genome of the American individual was assembled with GetOrganelle with default settings using the *G. mangostana* (OM759996) mitochondrial genome as seed. All FASTQ reads were used as input, as for the chloroplast genome assembly. The resulting scaffolds were aligned with the only available Clusiaceae mitochondrial assembly of *G. mangostana* (Wee et al. 2022) to retrieve the protein-coding genes.

### Nuclear genome characterization

The GenomeScope v2.0 web application (Ranallo-Benavidez et al. 2020, available at http://genomescope.org/genomescope2.0) was used to analyze genome characteristics and estimate the proportion of heterozygosity. It was also used to visualize the *k*-mer plot. The genome size was estimated by implementing *k*-mer counting methods. For the purposes of comparison, both Jellyfish v2.3.0 (Marçais and Kingsford 2011) and KMC v3.2.0 (Kokot et al. 2017) were used for *k*-mer count, setting the number of *k*-mers to 21. Max k-mer coverage was set in Jellyfish and GenomeScope to 1,000,000 instead of the default 10,000 as recommended to repetitive and highly heterozygous genomes.

### Nuclear genome assembly and annotation

Platanus-allee v2.2.2 (Kajitani et al. 2019) was used for the genome assembly as a suitable assembler for highly heterozygous genomes, although optimally recommended to be used with mate-pair libraries and >80% genomic coverage. A metric of gene completeness of the assembly was determined with BUSCO v5.4.7 using both the eudicots_ odb10 and embryophyta_odb10 databases (Manni et al. 2021). In addition, quality metrics were obtained with QUAST-LG (Mikheenko et al. 2018) and BUSCO v5.4.7 (Manni et al. 2021).

Functional annotation was done using the TOA (Taxonomy-oriented Annotation) pipeline (Mora-Márquez et al. 2021) in the mode of amino acid annotation. TOA is a transcriptome annotation platform with a focus on plants. Information from genomic databases were used sequentially in this order: Gymno PLAZA 1.0, Dicots PLAZA 4.0, Monocots PLAZA 4.0, NCBI RefSeq Plantmand, and NCBI Nucleotide Database (NT, selecting plant sequences), complemented with information from NCBI Gene, InterPro, and Gene Ontology databases as implemented in the pipeline. Taxonomic annotation with TOA detected 38 amino acids other than plants as first hit organisms. As the corresponding scaffolds were short (length between 47 and 281 bp), separate scaffolds, they were removed from the assembly before evidence-based prediction as likely contaminations.

The assembly was softmasked using the RepeatMasker v2.0.4 (Smit et al. 2013–2015) pipeline using the default NCBI search engine in RepeatModeler (Smit and Hubley 2008–2015). Simple reads were left unmasked, as they are frequently part of genes. The masked genome assembly was submitted to ENA with accession number GCA_965614385.1. Evidence-based gene prediction in the masked genome assembly was performed using the BRAKER2 pipeline, applying amino acid data as reference.

### Intraspecific genetic diversity

To estimate the number of differences between two geographically distant individuals, Transcriptome Ortholog Alignment Sequence Tools (TOAST; Wcisel et al. 2020) was used to retrieve the cds of orthologous single copy genes identified by BUSCO from both the African and American individual. The complete genes present in both individuals were concatenated with the Perl script seqCat.pl v1.0 (Bininda-Emonds 2005) into a 448,390 bp long concatenated matrix. The corresponding coding nucleotide sequences were extracted and aligned with MAFFT (Katoh and Standley 2013) with default settings and trimmed with trimAl v1.4.rev15 (Capella-Gutiérrez et al. 2009) using the option –strict. Alignment statistics for each orthogroup were obtained with SeqState (Müller 2005).

## Results and discussion

### Chloroplast genome assembly of African and American individuals

The de novo assembly of mate-pair (long) reads from the African individual using the A5 pipeline yielded 14 scaffolds. Aligning the scaffolds with the *Garcinia* chloroplast revealed that the assembled contigs did not cover the entire organelle. Therefore, a complete reconstruction of the chloroplast was not attempted. Instead, the coding sequences were retrieved for intraspecific comparison.

**Fig. 1.**
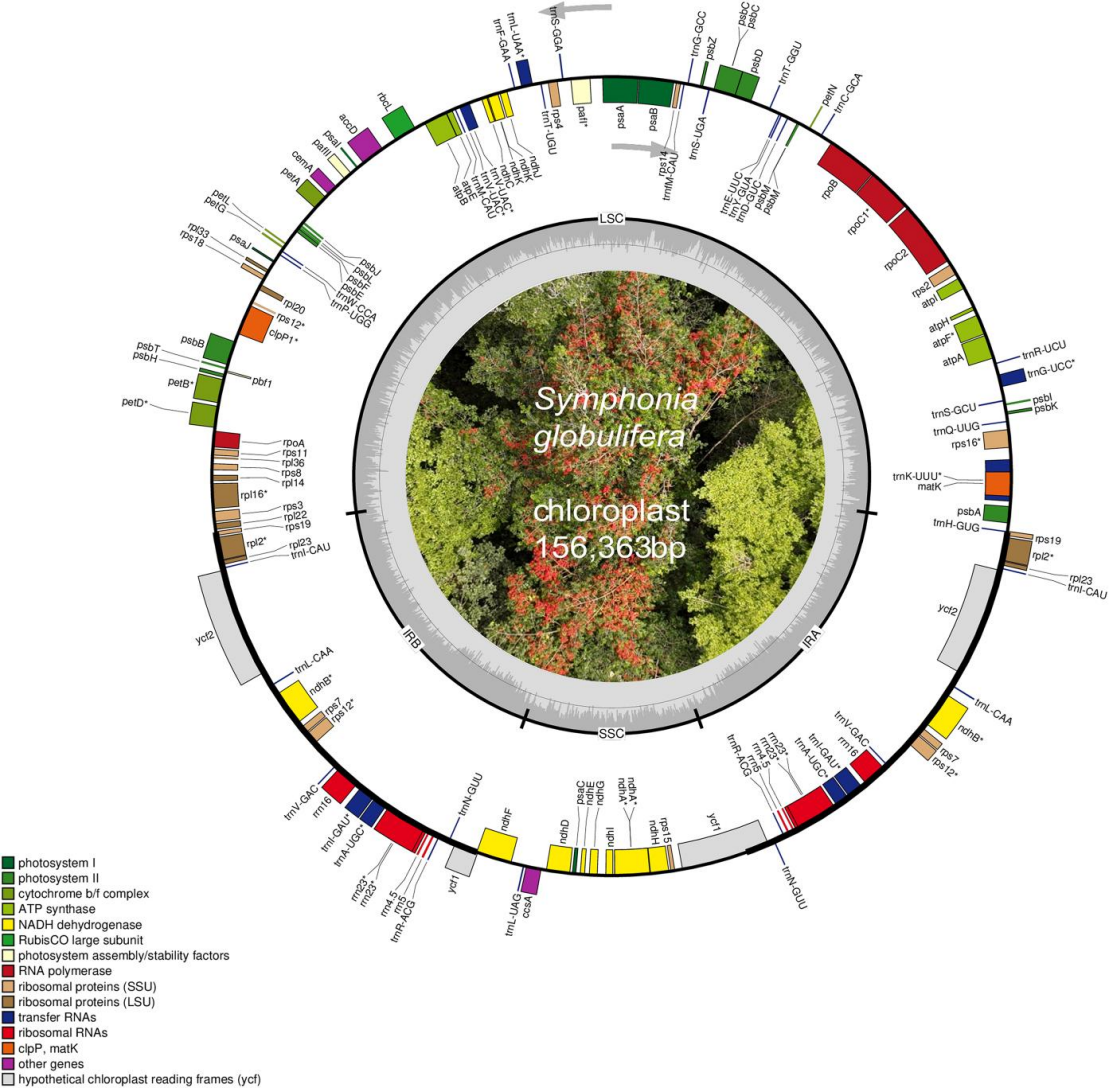
The circular plastome of *S. globulifera* (American individual). Genes inside the circle are transcribed clockwise, while genes outside the circle are transcribed anti-clockwise, as indicated by the gray arrows. The gray bars inside the circle represent the GC content of the sequence. Asterisks (*) indicate genes containing intron(s).

In contrast, the assembly based on Illumina reads of the American individual yielded a more complete plastome. De novo assembly using GetOrganelle resulted in five scaffolds (Supplementary Fig. 13). The average coverage of the chloroplast genome assembly with Illumina reads was 55×. Aligning against *Garcinia* sequences allowed us to reconstruct an almost complete 156,363 bp long circular plastome of *S. globulifera*, including a LSC (85,173 bp) and SSC (17,102 bp) separated by 27,044-bp-long inverted repeat (IR) regions (Fig. 1). However, six gaps remained in the repeat-rich intergenic regions of the assembly, indicated in the complete chloroplast assembly with 12 Ns (the exact number of Ns being unknown). The total number of missing characters was estimated to be 1,831 bp, based on the alignment with *Garcinia* species.

A total of 128 genes were annotated, of which 77 were unique protein-coding genes with six duplicated genes, 30 unique tRNAs (seven duplicated genes), and 4 rRNAs (four duplicated genes). All duplicated genes were located in the IR region. The chloroplast gene content and order were conserved in comparison with *Garcinia* species (Wee et al. 2023). Due to the availability of these closely related *Garcinia* chloroplast genomes, the rather modest amount of Illumina reads, together with long-read resources from another individual, allowed us to reconstruct an almost complete chloroplast genome of the species and to analyze intraspecific variation. Photo credit: Benoit Burban.

### Phylogenetic relationships of *Symphonia* and *Garcinia* based on chloroplast genes

**Fig. 2.**
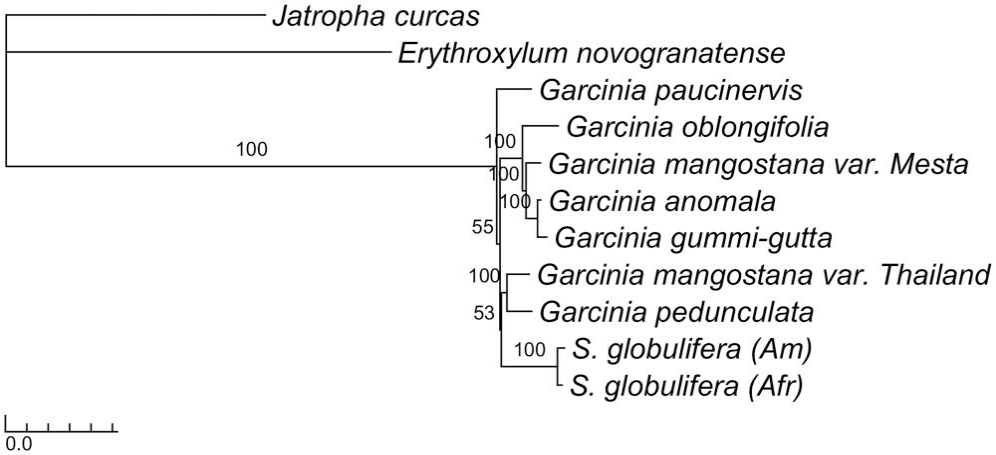
Phylogenetic relationships of *S. globulifera* in relation to *Garcinia* based on 77 coding chloroplast genes. The support values labeled at each node indicate bootstrapped ML values. *J. curcas* and *E. novogranatense* served as outgroups.

The alignment consisting of 77 protein-coding chloroplast genes of the two *S. globulifera* and seven *Garcinia* individuals, together with the two outgroup species (*J. curcas* and *E. novogranatense*), was 82,086 bp long. All genes were retrieved from all specimens, except for *rps16*, which was not present in the outgroup species (Supplementary Table 1). The highest number of indels was observed in the ycf3_exon2 region. The phylogenetic reconstruction shows a fully supported *Garcinia*–*Symphonia* clade (Fig. 2). The internal nodes are mostly fully supported, with two exceptions: neither the position of *Garcinia paucinervis* within the *Garcinia*–*Symphonia* clade (bootstrap support 55) nor the sister clade relationship of *S. globulifera* with *G. mangostana* var. Thailand and *G. pedunculata* (bootstrap support 53) is well supported.

According to phylogenetic studies (Sweeney 2008, based on two nuclear markers; Gaudeul et al. 2024, based on one nuclear and three chloroplast markers), *Garcinia* is sister genus to *Symphonia*. Our analyses suggest that *Garcinia* is potentially paraphyletic as *S. globulifera* forms a clade with *G. pedunculata* and *G. mangostana* var. Thailand. The nonmonophyly of *G. mangostana* lineages based on chloroplast sequences has been described by Wee et al. (2023). However, these results were based on a limited number of species, as is the case with our study. In addition, biological phenomena like incomplete lineage sorting, hybridization, limited marker resolution, or chloroplast capture could also explain why *S. globulifera* groups together with some *Garcinia* species. The recent study by Gaudeul et al. (2024) suggests a broad circumscription of *Garcinia* to include *Allanblackia* and some other previously described genera, but before knowing if *Symphonia* should be included in *Garcinia*, or any other taxonomic changes, a more thorough phylogenetic study is based on a comprehensive taxon selection and, ideally, including also nuclear single-copy genes. The chloroplast genome and manually curated chloroplast gene alignments will be an important resource to study phylogenetic relationships of *Symphonia* and *Garcinia*. The same applies to the nuclear single-copy genes identified in this study. These genes form the basis for designing and testing primers that can be used to amplify them in a larger number of species.

### Mitochondrial genome assembly and annotation

The de novo assembly resulted in 14 scaffolds of which 8 were selected. The challenges of mitochondrial assembly are well known (as explained in the Introduction), and the short-read data alone did not yield a reasonable de novo assembly. However, when aligning the eight scaffolds against the *Garcinia* reference, all the same protein-coding genes as in *G. mangostana* were retrieved (Supplementary Fig. 2), which will be an additional resource for evolutionary studies. A comparison of these genes between the two species shows a very low level of differences (Supplementary Table 2).

### Nuclear genome characterization

The sequencing of 12 libraries yielded a total of a bit more than one billion reads. The genome was estimated to have high heterozygosity (1.62 to 1.69%) and low coverage (×16.6). According to GenomeScope (Vurture et al. 2017), a heterozygosity rate above 1.5% is considered high. The genome size was estimated by Jellyfish to be between 1.25 and 1.26 Gbp, of which unique sequences 411 to 414 Mbp and read error rate 0.10%. KMC estimated a smaller genome size, 0.95 −0.96 Gbp, and higher read error rate (0.14%). The first peak in GenomeScope profile was lower than the second one, which is probably due low coverage and high heterozygosity (Supplementary Fig. 3). The profile also showed a lower peak at around 90× coverage, which was interpreted as high coverage organellar reads, as the removal of organellar reads was incomplete.

### Nuclear genome assembly and annotation

**Fig. 3.**
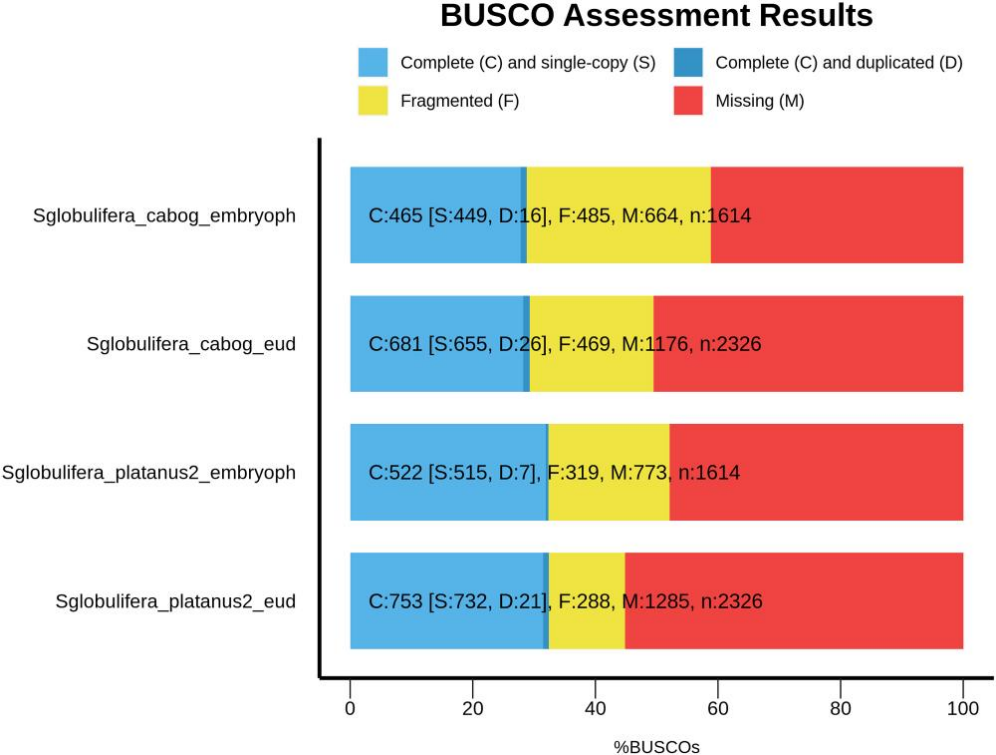
Completeness of the assembled genome (Sglobulifera_platanus2) and for comparison, the existing genome assembly of an African individual (Sglobulifera_cabog). The databases used were the eudicots_ odb10 (eud) and embryophyta_odb10 (embryoph).

The assembly had a N50 = 8,971 and yielded 72,765 contigs of which 8,037 were longer than 10,000 bp and 14 longer than 50,000 bp (Supplementary Table 3). The number of long scaffolds and BUSCO completeness was slightly better than the existing draft genome of the species (Olsson et al. 2017, comparison in Fig. 3), based on the number of complete genes. There were 522 (32.3%) complete (compared to 465 in earlier genome) and 319 (19.8%) fragmented genes when compared against the embryophyta_odb10 database, while 753 (32.4%) complete (compared to 681 in earlier) and 288 (12.4%) fragmented genes when compared against the eudicots_ odb10 database. Despite not meeting the optimal conditions for genome assembly with the chosen assembler, the produced genome assembly is slightly better than the previously available draft genome from an African individual.

The RepeatMasker pipeline masked 490,173 repeats totaling 166,387,956 bp. RepeatScout found 2,285 families. However, these results should be considered with caution as the N50 of the assembly was low (<10,000 bp) and consequently RepeatModeler may not perform well with short contigs (Smit 2008–2015). The BRAKER2 annotation pipeline resulted in 79,619 coding genes, which were made available on Zenodo (DOI: 10.5281/zenodo.13270663), together with the genome assembly and annotation file.

### Intraspecific genetic diversity

A total of 1,131 complete BUSCO single copy genes were retrieved from the *S. globulifera* genomes, of which 256 were present in both the African and American individual. Genetic diversity was measured in these shared 256 nuclear orthologous single-copy genes, which yielded a 448,390-bp-long concatenated alignment. Great variability in the length could be observed as the trimmed alignment (without introns present in only one of the specimens and without spurious sequences and poorly aligned regions) was only 161,212 bp long, of which 6.14% were variable.

It has become clear that one reference genome per species is not enough to capture the intraspecific genetic diversity in plants, as individuals of the same species often exhibit significant differences in the genome size, structural variants, and nucleotide-level polymorphisms such as SNPs and indels (Bayer et al. 2020). In addition to economically important species, genomic information from multiple individuals will be needed for tropical species to advance research on them, especially in species with high levels of intraspecific variation such as *S. globulifera*.

This high intraspecific genetic diversity has important implications for evolutionary biology and conservation. It suggests a rich genomic substrate for adaptation to diverse environments and may reflect complex demographic histories, including historical isolation, migration, or hybridization (Zuellig et al. 2014). From a conservation standpoint, recognizing and preserving such diversity is crucial for maintaining the species' evolutionary potential, especially under changing climate conditions.

## Conclusions

This study provides the first comprehensive genome-wide comparison between African and South American individuals of *S. globulifera*, including de novo assembled nuclear, chloroplast, and mitochondrial genomes. The results reveal significant intraspecific genomic variation, underscore the importance of genomic resources in tropical species, and point out the need for a comprehensive phylogenetic study of relationships between *Symphonia* and *Garcinia*. The new genomic resources, including annotated genes and curated alignments, will be instrumental for future studies in tropical phylogenetics, comparative genomics, and conservation biology. Together, these findings highlight the need to include multiple individuals in plant genome projects to fully capture the diversity and evolutionary history within widespread tropical species.

## Supplementary Material

jkaf208_Supplementary_Data

## Data Availability

The raw reads, nuclear genome assembly (GCA_965614385.1), and chloroplast assembly (OZ172679.1) are available from the ENA under project PRJEB62664. Nuclear coding sequences from the de novo genome assembly, the annotated genome assembly, and pairwise alignments of nuclear single-copy genes between African and American *S. globulifera* specimens are available from Zenodo under DOI 10.5281/zenodo.13270663 together with aligned chloroplast and mitochondrial coding genes. Supplemental material available at G3 online.
