# Supplementary material for: De novo assembled nuclear, chloroplast, and mitochondrial genomes show high intraspecific variation in the tropical rainforest species Symphonia globulifera
Source: G3 (Bethesda). 2025 Sep 10;15(11):jkaf208. doi: 10.1093/g3journal/jkaf208 (PMC12611251; doi:10.1093/g3journal/jkaf208)
Supplement: jkaf208_Supplementary_Data [file jkaf208_supplementary_data.pdf]

SUPPLEMENTARY MATERIAL

Supplementary Figure S1. Five selected *Symphonia globulifera* de novo assembled chloroplast scaffolds assembled using GetOrganelle and visualized in Bandage.

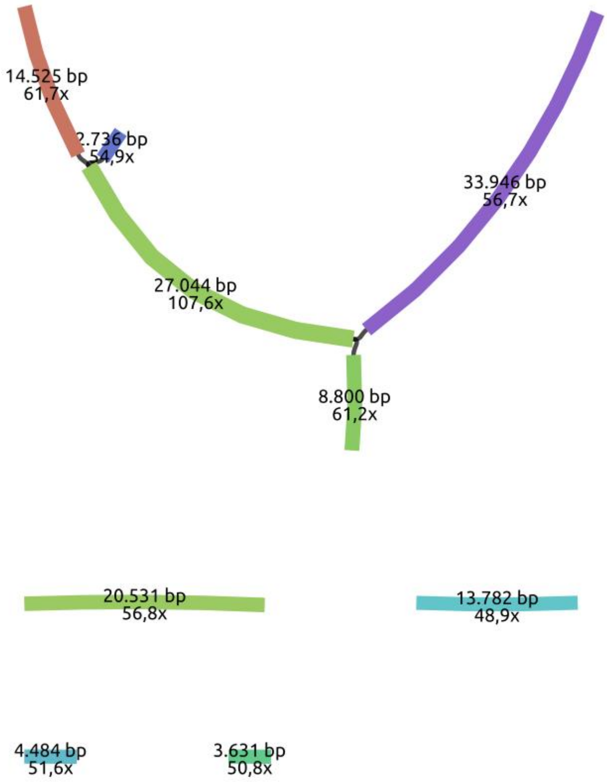

Supplementary Figure S2. The *de novo* assembled mitochondrial genome of *Symphonia globulifera*. The assembly was incomplete and length estimation is based on aligning assembled scaffolds against Garcinia mangostana mitochondrial genome. Genes inside the circle are transcribed clockwise while genes outside the circle are transcribed anti-clockwise, as indicated by the gray arrows. The gray bars inside the circle represent the GC content of the sequence. Asterisks (*) indicate genes containing intron(s). Photo credit: Benoit Burban.

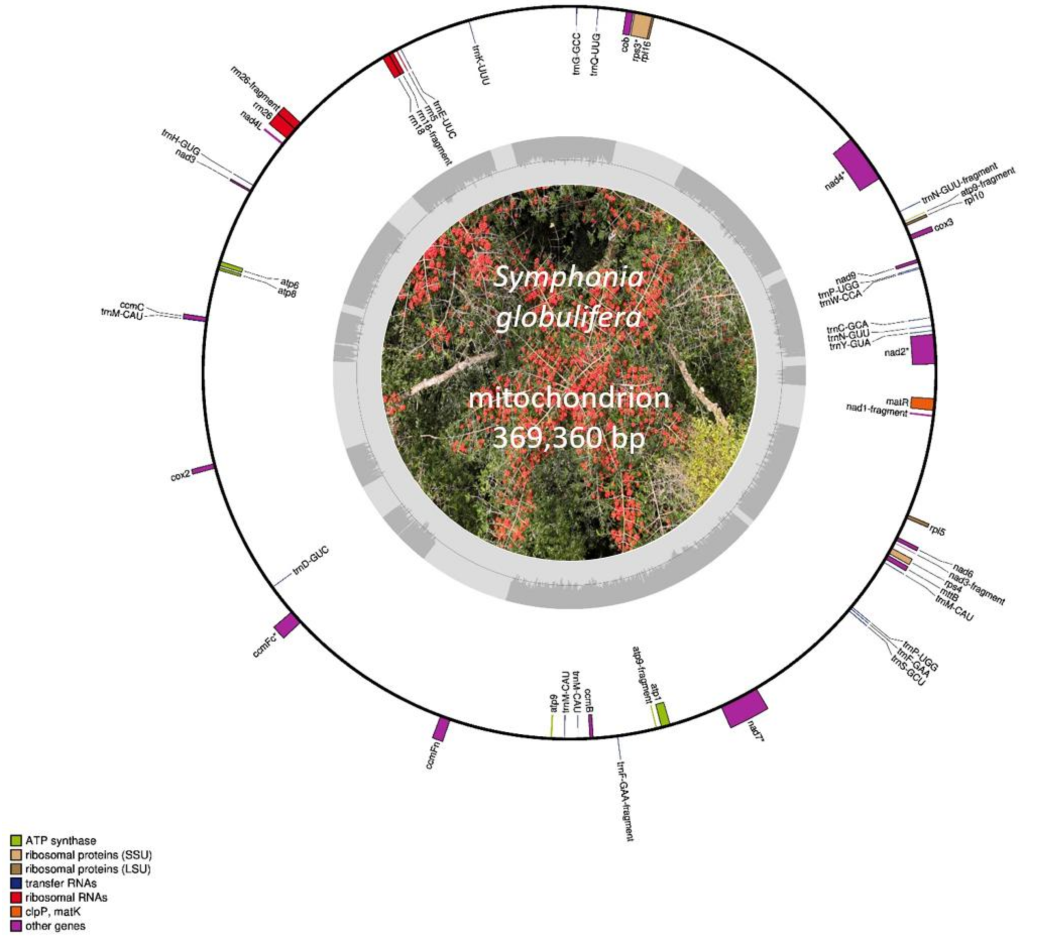

Supplementary Figure S3. GenomeScope profile of *Symphonia globulifera* with k=21.

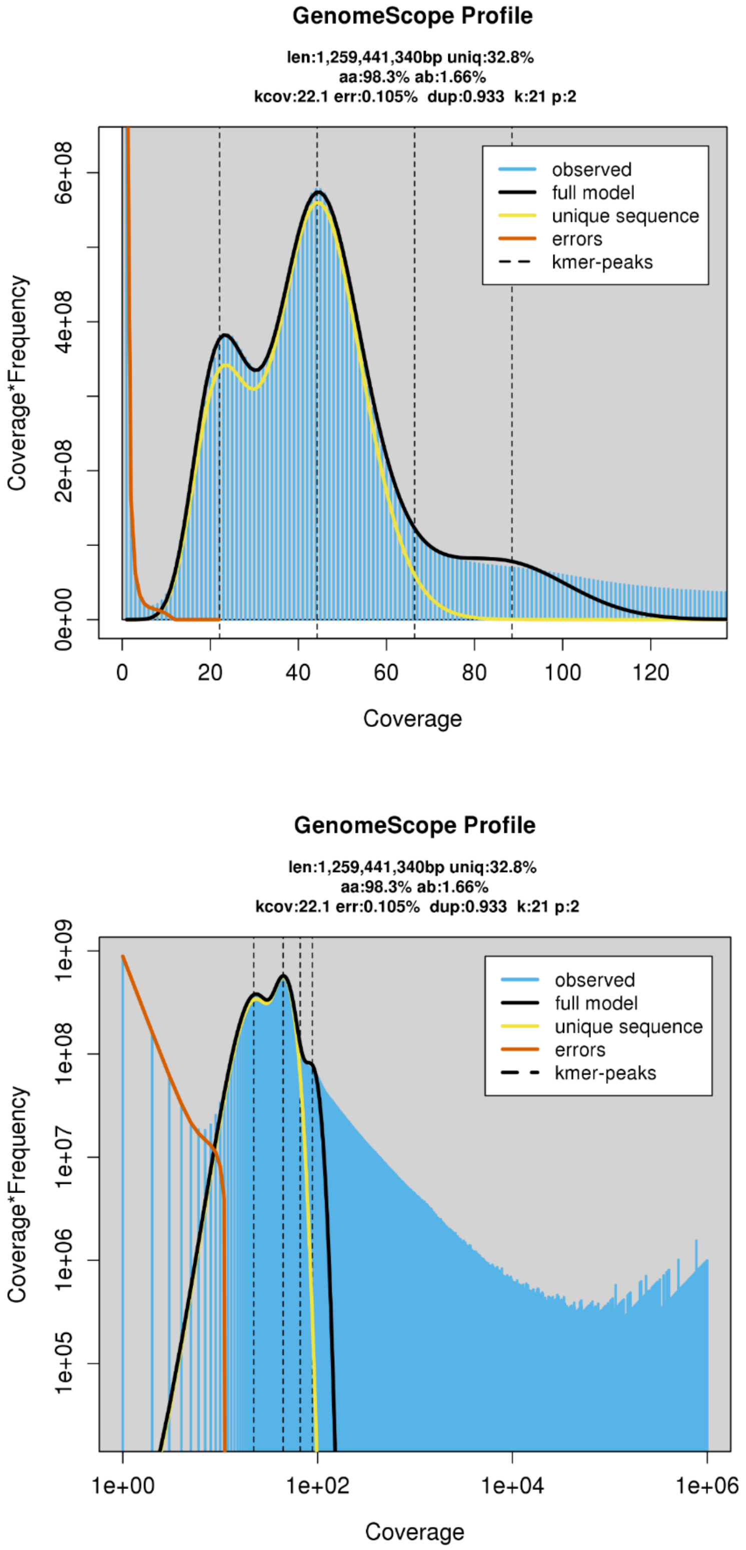

Supplementary Table S1. Length (bp) and number of indels of the annotated 77 protein coding chloroplast gene alignments used in the phylogenetic reconstruction. For genes with introns, the length is given for each gene feature. The two *Symphonia globulifera* individuals (*S. globulifera* Am = America; *S. globulifera* Afr. = Africa) were aligned together with seven *Garcinia* species as well as *Jatropha curcas* and *Erythroxylum novogranatense* as outgroups. Gene *rps12** is a trans-spliced gene with 5'end located at the LSC regions while the duplicated 3' ends located at the IR regions.

	Jatropha curcas	*Garcinia paucinervis*	*Garcinia anomala*	*Garcinia mangostana* var. Mesta	*Erythroxylum novogranatense*	*Garcinia mangostana* var. Thailand	*Garcinia gummi-gutta*	*Garcinia pedunculata*	*Garcinia oblongifolia*	*S. globulifera* Am (this study)	*S. globulifera* Afr (this study)
ENA accession numbers	NC 012224	MT501656	MW582313	MZ823408	NC 030601	NC 036341	NC 047250	NC 048983	NC 050384	ERZ24825350	NA
atpA	1524	1533	1533	1533	1524	1533	1533	1533	1533	1533	1533 (18 indels)
atpB	1497 (9 indels)	1497	1497	1497	1497	1497	1497	1497	1497	1497	1497 (18 indels)
atpE	408 (6 indels)	408	408	408	408 (6 indels)	408	408	408	408	408	408
atpH	246	246	246	246	246	246	246	246	246	246	246 (15 indels)
atpI	744	744	744	744	744	744	744	744	744	744	744 (15 indels)
ccsA	993 (18 indels)	993 (21 indels)	993 (21 indels)	993 (21 indels)	993 (21 indels)	993 (21 indels)	993 (21 indels)	993 (21 indels)	993 (21 indels)	993 (30 indels)	993 (30 indels)
cemA	731 (5 indels)	731 (5 indels)	731 (14 indels)	731 (17 indels)	731 (3 indels)	731 (5 indels)	762 (15 indels)	731 (5 indels)	762 (12 indels)	762 (5 indels)	762 (5 indels)
atpF_exon1	145	145	145	145	145	145	145	145	145	145	145
atpF_intron	801 (76 indels)	801 (53 indels)	801 (40 indels)	801 (50 indels)	801 (82 indels)	801 (58 indels)	801 (41 indels)	801 (57 indels)	801 (40 indels)	801 (56 indels)	801 (63 indels)
atpF_exon2	365	398	398	398	398	398	398	398	398	398	398
clpP_exon1	71	71	71	71	71	71	71	71	71	71	71
clpP_intron1	1002 (139 indels)	1002 (257 indels)	1002 (249 indels)	1002 (247 indels)	1002 (132 indels)	1002 (266 indels)	1002 (254 indels)	1002 (246 indels)	1002 (251 indels)	1002 (263 indels)	1002 (260 indels)
clpP_exon2	292	292	292	292	292	292	292	292	292	292	292
clpP_intron2	759 (106 indels)	759 (118 indels)	759 (146 indels)	759 (147 indels)	759 (75 indels)	759 (122 indels)	759 (146 indels)	759 (111 indels)	759 (130 indels)	759 (154 indels)	759 (157 indels)

clpP_exon3	228	228	228	228	228	228	228	228	228	228	228
matK	1605 (75 indels)	1611 (63 indels)	1611 (57 indels)	1611 (63 indels)	1611 (54 indels)	1611 (63 indels)	1611 (57 indels)	1611 (69 indels)	1611 (63 indels)	1611 (63 indels)	762 (48 indels)
ndhC	363	363	363	363	363	363	363	363	363	363	363
ndhD	1503	1503	1503	1503	1503	1503	1503	1503	1503	1503	1503 (12 indels)
ndhE	306	306	306	306	303	306	306	306	306	306	306
ndhF	2442 (75 indels)	2442 (171 indels)	2442 (171 indels)	2442 (171 indels)	2313 (51 indels)	2442 (168 indels)	2442 (171 indels)	2442 (168 indels)	2442 (156 indels)	2442 (183 indels)	1985 (91 indels)
ndhG	531	531	531	531	531	531	531	531	531	531	531
ndhH	1188 (6 indels)	1188 (6 indels)	1188 (6 indels)	1188 (6 indels)	1188	1188 (12 indels)	1188 (6 indels)	1188 (6 indels)	1188 (6 indels)	1188 (6 indels)	1188 (6 indels)
ndhI	522 (9 indels)	522 (45 indels)	522 (45 indels)	522	522 (24 indels)	522 (45 indels)	522 (36 indels)	522 (45 indels)	522 (45 indels)	522 (45 indels)	522 (45 indels)
ndhA_exon1	567 (15 indels)	567 (6 indels)	567 (6 indels)	567 (6 indels)	558 (6 indels)	567 (6 indels)	567 (6 indels)	567 (6 indels)	567 (6 indels)	567 (6 indels)	567 (6 indels)
ndhA_intron	1416 (281 indels)	1416 (268 indels)	1416 (282 indels)	1416 (259 indels)	1416 (219 indels)	1416 (259 indels)	1416 (280 indels)	1416 (276 indels)	1416 (330 indels)	1416 (283 indels)	1416 (275 indels)
ndhA_exon2	546 (6 indels)	546 (12 indels)	546 (12 indels)	546 (12 indels)	546 (9 indels)	546 (12 indels)	546 (12 indels)	546 (12 indels)	546 (12 indels)	546 (12 indels)	546 (12 indels)
ndhB_exon1	777	777	777	777	777	777	777	777	777	777	777
ndhB_intron	706 (24 indels)	706 (7 indels)	706 (7 indels)	706 (9 indels)	706 (19 indels)	706 (8 indels)	706 (7 indels)	706 (5 indels)	706 (6 indels)	706 (9 indels)	706 (16 indels)
ndhB_exon2	756 (6 indels)	756	756	756	756	756	756	756	756	756	756
ndhJ	477	477	477	477	477	477	477	477	477	477	477 (21 indels)
ndhK	705 (27 indels)	705 (27 indels)	705 (27 indels)	705 (27 indels)	705	705 (27 indels)	705 (27 indels)	705 (27 indels)	705 (27 indels)	705 (27 indels)	705 (27 indels)
petA	963	963	963	963	963	963	963	963	963	963	963
petG	114	114	114	114	114	114	114	114	114	114	114
petL	102 (6 indels)	102 (6 indels)	102 (6 indels)	102 (6 indels)	102 (6 indels)	102	102 (6 indels)	102 (6 indels)	102 (6 indels)	102 (6 indels)	102 (6 indels)
petN	90	90	90	90	90	90	90	90	90	90	90 (13 indels)
psaA	2253	2253	2253	2253	2253	2253	2253	2253	2253	2253	2253
psaB	2205	2205	2205	2205	2205	2205	2205	2205	2205	2205	2205 (35 indels)
petB_exon1	6	6	6	6	6	6	6	6	6	6	6
petB_intron	902 (124 indels)	902 (61 indels)	902 (50 indels)	902 (64 indels)	902 (81 indels)	902 (73 indels)	902 (51 indels)	902 (98 indels)	902 (74 indels)	902 (56 indels)	902 (57 indels)
petB_exon2	641	641	641	641	641	641	641	641	641	641	641

petD_exon1	8	8	8	8	8	8	8	8	8	8	8
petD_intron	1046 (185 indels)	23 (1 indels)	1046 (226 indels)	1046 (248 indels)	1046 (224 indels)	1046 (233 indels)	1046 (234 indels)	1046 (239 indels)	1046 (248 indels)	1046 (176 indels)	1046 (206 indels)
petD_exon2	496	526	526	526	526 (1 indels)	496	526	526	526	526	526 (12 indels)
psaC_fasta	246	246	246	246	246	246	246	246	246	246	246
psaI	114 (1 indels)	114	114	114	114	114	114	114	114	114	114
psaJ	135	135	135	135	135	135	135	135	135	135	135
psbA	1062	1062	1062	1062	1062	1062	1062	1062	1062	1062	1062
psbB	1527	1527	1527	1527	(No data)	1527	1527	1527	1527	1527	1527 (6 indels)
psbC	1422	1422	1422	1422	1422	1422	1422	1422	1422	1422	1422 (7 indels)
psbD	1062	1062	1062	1062	1062	1062	1062	1062	1062	1062	1062
psbE	252	252	252	252	252	252	252	252	252	252	252
psbF	120	120	120	120	120	120	120	120	120	120	120
psbH	222	222	222	222	222	222	222	222	222	222	222
psbI	111	111	111	111	111	111	111	111	111	111	111
psbJ	123	123	123	123	123	123	123	123	123	123	123
psbK	195	186	186	186	186	186	186	186	186	186	186
psbL	117	117	117	117	117	117	117	117	117	117	117
psbM	114 (9 indels)	114 (9 indels)	114 (9 indels)	114 (9 indels)	114	114 (9 indels)	114 (9 indels)	114 (9 indels)	114 (9 indels)	114 (9 indels)	114 (9 indels)
psbN	132	132	132	132	132	132	132	132	132	(No data)	(No data)
psbT	108	108	108	108	108	108	108	108	108	108	108
psbZ	189	189	189	189	(No data)	189	189	189	189	189	189
rbcL	1437 (9 indels)	1437 (9 indels)	1437 (9 indels)	1437 (9 indels)	1437 (9 indels)	1437	1437 (9 indels)	1437 (9 indels)	1437 (9 indels)	1437 (9 indels)	1437 (9 indels)
rpl14	369	369	369	369	369	369	369	369	369	369	369 (16 indels)
rpl20	420 (60 indels)	420 (66 indels)	420 (57 indels)	420 (66 indels)	420 (18 indels)	420 (66 indels)	420 (66 indels)	420 (66 indels)	420 (6 indels)	420 (66 indels)	420 (86 indels)
rpl22	504 (111 indels)	504 (144 indels)	504 (111 indels)	504 (78 indels)	504 (6 indels)	504 (111 indels)	504 (126 indels)	504 (111 indels)	504 (111 indels)	504 (105 indels)	504 (105 indels)
rpl23	282	282	282	282	282	282	282	282	282	282	282

rpl33	201	201	201	201	201	201	201	201	201	201	201
rpl36	114	114	114	114	114	114	114	114	114	114	114
rpoA	1020	1020	1020	1020	1020	1020	1020	1020	1020	1020	1020 (29 indels)
rpoB	3228 (15 indels)	3228 (15 indels)	3228 (15 indels)	3228 (9 indels)	3225 (15 indels)	3228 (15 indels)	3228 (15 indels)	3228 (15 indels)	3228 (9 indels)	3228 (6 indels)	3228 (49 indels)
rpl12_exon1	405 (5 indels)	405 (5 indels)	405 (5 indels)	405 (14 indels)	405 (14 indels)	405 (5 indels)	405 (5 indels)	405 (5 indels)	405 (5 indels)	405 (5 indels)	405
rpl12_exon2	434	434	434	434	434	434	434	434	434	434	434
rpl12_intron	764 (95 indels)	764 (103 indels)	764 (103 indels)	764 (96 indels)	764 (97 indels)	764 (103 indels)	764 (103 indels)	764 (103 indels)	764 (95 indels)	764 (103 indels)	764 (21 indels)
rpl16_exon1	9	9	9	9	9	9	9	9	9	9	9
rpl16_intron	(No data)	1254 (140 indels)	1254 (95 indels)	(No data)	(No data)	1254 (124 indels)	1254 (160 indels)	1254 (127 indels)	1254 (112 indels)	1254 (136 indels)	1254 (104 indels)
rpl16_exon2	399	399	399	399	399	399	399	399	399	399	399
rpoC1_exon1	438 (6 indels)	438 (6 indels)	438 (6 indels)	438 (6 indels)	459	438 (6 indels)	438 (6 indels)	438 (6 indels)	438 (6 indels)	438 (6 indels)	438 (6 indels)
rpoC1_intron	870 (117 indels)	870 (109 indels)	870 (115 indels)	870 (90 indels)	870 (104 indels)	870 (117 indels)	870 (101 indels)	870 (105 indels)	870 (112 indels)	870 (108 indels)	870 (97 indels)
rpoC1_exon2	1647 (15 indels)	1647 (15 indels)	1647 (12 indels)	1626 (15 indels)	1647 (12 indels)	1647 (15 indels)	1647 (15 indels)	1647 (15 indels)	1647 (15 indels)	1647 (17 indels)	1647 (17 indels)
rpoC2	4221 (36 indels)	4221 (69 indels)	4221 (57 indels)	4221 (57 indels)	4209 (96 indels)	4221 (42 indels)	4221 (57 indels)	4221 (63 indels)	4221 (57 indels)	(No data)	4209 (71 indels)
rps11	423 (6 indels)	423 (6 indels)	423 (6 indels)	423 (6 indels)	423	423 (6 indels)	423 (6 indels)	423 (6 indels)	423 (6 indels)	423 (6 indels)	423 (6 indels)
rps14	303	303	303	303	303	303	303	303	303	303	303
rps15	279 (6 indels)	279	279 (6 indels)	279 (6 indels)	279 (15 indels)	279 (6 indels)	279 (3 indels)	279 (6 indels)	279 (6 indels)	279 (6 indels)	279 (6 indels)
rps2	711	711	711	711	711	711	711	711	711	711	711
rps3	681 (15 indels)	681 (24 indels)	681 (24 indels)	681 (24 indels)	681 (9 indels)	681 (24 indels)	681 (24 indels)	681 (24 indels)	681 (24 indels)	681 (24 indels)	681 (24 indels)
rps4	606	606	606	606	606	606	606	606	606	606	606
rps7	476 (8 indels)	476 (8 indels)	476 (8 indels)	476 (8 indels)	476 (7 indels)	476 (8 indels)	476 (8 indels)	476 (8 indels)	476 (8 indels)	476 (8 indels)	476 (8 indels)
rps8	417 (12 indels)	417 (12 indels)	417 (12 indels)	417 (12 indels)	417 (12 indels)	417 (12 indels)	417 (12 indels)	417 (12 indels)	417 (12 indels)	417	417
rps12_exon1	114	114	114	114	114	114	114	114	114	114 (11 indels)	114
rps12_exon2	232	232	232	232	232	232	232	232	232	232	232
rps12_exon3*	26	26	26	26	26	26	26	26	26	26	26

rps12_intron	553 (8 indels)	553 (15 indels)	553 (15 indels)	553 (15 indels)	553 (11 indels)	553 (15 indels)	553 (15 indels)	553 (15 indels)	553 (15 indels)	553 (15 indels)	553 (15 indels)
rps18	327	306	306	306	323 (5 indels)	306	306	306	306	306	306
rps19	279	279	279	279	279	279	279	279	279	279	279
ycf1	5900 (176 indels)	5903 (239 indels)	5903 (248 indels)	5903 (254 indels)	5903 (236 indels)	5903 (278 indels)	5903 (245 indels)	5903 (257 indels)	5903 (248 indels)	5903 (299 indels)	5903 (93 'N', 337 indels)
ycf2	7002 (105 indels)	7005 (126 indels)	7005 (90 indels)	7005 (108 indels)	7002 (45 indels)	7005 (108 indels)	7005 (90 indels)	7005 (114 indels)	7005 (90 indels)	7005 (114 indels)	7005 (168 indels)
ycf4	555	555	555	555	555	555	555	555	555	555	555
rps16_exon1	(No data)	36	36	36	(No data)	36	36	36	36	36	36
rps16_intron	(No data)	792 (16 indels)	943 (38 indels)	943 (35 indels)	(No data)	943 (20 indels)	943 (33 indels)	943 (19 indels)	(No data)	943 (55 indels)	943 (63 indels)
rps16_exon2	(No data)	230 (5 indels)	189 (6 indels)	189 (6 indels)	(No data)	188 (5 indels)	189 (6 indels)	71 (5 indels)	121 (7 indels)	237 (7 indels)	237 (7 indels)
ycf3_exon1	126	126	126	126	126	126	126	126	126	126	126
ycf3_intron	753 (59 indels)	753 (24 indels)	753 (36 indels)	753 (31 indels)	753 (25 indels)	753 (28 indels)	753 (35 indels)	753 (31 indels)	753 (31 indels)	753 (33 indels)	753 (55 indels)
ycf3_exon2	1129 (65 indels)	1129 (742 indels)	1129 (742 indels)	1129 (742 indels)	1129 (16 indels)	1129 (742 indels)	1129 (742 indels)	1129 (742 indels)	1129 (742 indels)	1129 (748 indels)	1129 (748 indels)

Supplementary Table S2. Length (bp) and number of indels of the annotated 28 protein coding mitochondrial gene alignments. For genes with introns, the length is given for each gene feature. The *de novo* assembled *Symphonia globulifera* mitochodrium scaffolds were aligned together with *Garcinia mangostana* (OM759996.1).

	Symphonia globulifera	*Garcinia mangostana*
atp1	1506	1512
atp4	612	612
atp6	768	768
atp8	474	474
atp9	225 (4 'N', 1 indels)	225
ccmB	621	621
ccmC	753	753
ccmFc_exon1	762	762
ccmFc_intron	579	579
ccmFc_exon2	934 (11 indels)	934
ccmFn	1722	1720 (6 indels)
cox2	798	798
cox3	795	803
matR	1959	1959
mttB	780	780
nad1	978	259
nad2_exon1	1920	161

	Symphonia globulifera	*Garcinia mangostana*
nad2_intron1	(No data)	2525
nad2_exon2	573	573
nad2_intron2	(No data)	1421
nad2_exon3	188	188
nad3	357	357
nad4	7943 (3 indels)	7943 (43 indels)
nad4L	303	303
nad5_exon1	224	224
nad5_intron1	842	842
nad5_exon2	1223	1223
nad5_intron2	21	21
nad5_exon3	394	394
nad5_intron3	1073	1073
nad5_exon4	151	151
nad6	618	618
nad9	573	573
nad7_exon1	143	143
nad7_intron1	892	892
nad7_exon2	69	69
nad7_intron2	1521 (1 indel)	1521

	Symphonia globulifera	*Garcinia mangostana*
nad7_exon3	466	466
nad7_intron3	1045 (1 indel)	1045
nad7_exon4	244	244
nad7_intron4	1760	1760
nad7_exon5	263	263
rpl10	441	441
rpl16	447	447 (8 indels)
rpl5	615	615 (5 indels)
rps3_exon1	74	74
rps3_intron	1509 (4 indels)	1509 (1 indel)
rps3_exon2	1411	1411
rps4	1026	1026

Supplementary Table S3. Statistics on the assembly produced with QUAST-LG. A) Number of contigs of different lengths. B) Assembly quality indicators based on contig lengths. All statistics were based on contigs of size >= 3000 bp. N50 and N90 = are the lengths L of the shortest contig for which longer and equal length contigs cover at least 50% of the assembly, where auN is the area under the Nx curves. L50 = count of smallest number of contigs whose length sum makes up half of genome size. C) Other assembly measures.

A)

Size of contigs	Number of contigs	Length in bp
>= 0 bp	31,2240	399,084,445
>= 1,000 bp	72,765	348,269,027
>= 5,000 bp	22,462	226,217,463

>= 10,000 bp	8,037	125,387,585
>= 25,000 bp	564	17,446,697
>= 50,000 bp	14	774,335
Total number of contigs	37,265	

B)

Quality indicator	Length in bp
Largest contig	63,944
Total length	283,735,353
N50	8,971
N90	3,946
auN	11,219.4
L50	9,781
L90	29,020

C)

Assembly measure	
GC content (%)	36.3
N's per 100 kbp	96.98
